# Report of the Distribution Committee

**Published:** 1920-12-25

**Authors:** 


### KING EDWARD'S HOSPITAL FUND FOR LONDON.

### REPORT OF THE DISTRIBUTION COMMITTEE (*continued from p. 267*).

### Grants Recommended by the Committee.

Note 1.—*As foreshadowed in July last, the Distribution Committee, in arriving at the amount of the ordinary grant, have taken into consideration the amount of the emergency grant.*

Note 2.—*The Distribution Committee desire to draw attention to the fact that it must not be assumed that the reduction or absence of a grant implies dissatisfaction.*

Acton Hospital, £350, of which £250 to extension, in accordance with the scheme submitted to the Fund. Alexandra Hospital for Crippled Children, £100. Beckenham Cottage Hospital, £300, of which £250 to extension, in accordance with the scheme submitted to the Fund. Belgrave Hospital for Children, £1,000. Blackheath and Charlton Hospital, £150. Bolingbroke Hospital, £750. British Hospital for Mothers and Babies, Woolwich, £1,650, of which £1,500 towards the building of the amalgamated hospital, in accordance with the scheme submitted to the Fund. Canning Town Women's Settlement Hospital, £600, of which £100 to reduce debt. Central London Ophthalmic Hospital, £350. Central London Throat and Ear Hospital, £100. Charing Cross Hospital, £5,000, of which £250 to improvements. Chelsea Hospital for Women, £1,500, of which £1,000 to new nurses' home, in accordance with the scheme submitted to the Fund. Cheyne Hospital for Children, £150. City of London Hospital for Diseases of the Chest (Victoria Park), £3,000, of which £500 to proposed sanitary improvements, in accordance with the scheme submitted to the Fund. City of London Maternity Hospital, £600. Clapham Maternity Hospital, £400. Dreadnought Hospital (Seamen's), £4,000; the amount of the ordinary grant to this Hospital this year is being generously given by King George's Fund for Sailors, as a temporary measure, earmarked for the Seamen's Dreadnought Hospital. East End Mothers' Lying-in Home, £450. East Ham Hospital, £600, of which £500 to rebuilding and extension as a general hospital, in accordance with scheme to be submitted to the Fund. East London Hospital for Children, £1,500. Elizabeth Garrett Anderson Hospital, £1,000. Eltham and Mottingham Cottage Hospital, £100. Finchley Cottage Hospital, £600, of which £500 to extension, in accordance with the scheme submitted to the Fund. Florence Nightingale Hospital for Gentlewomen, £300, of which £100 to reduce debt. French Hospital, £200, of which £100 to improvements, in accordance with the scheme submitted to the Fund. General Lying-in Hospital, £1,400, of which £1,000 to rebuilding on new site, in accordance with scheme to be submitted to the Fund. Great Northern Central Hospital, £6,000, of which £2,000 to new nurses' home, in accordance with the scheme submitted to the Fund. Grosvenor Hospital for Women, £600. Guy's Hospital, £12,000, of which £1,000 to improvements in nursing accommodation, in accordance with the scheme submitted to the Fund. Hampstead General and North-West London Hospital, £2,600, of which £1,000 to reduce debt and £100 to improvements. Hendon Cottage Hospital, £300, of which £100 towards new operating theatre, $x$-ray and nurses' home improvements, in accordance with the scheme submitted to the Fund. Hornsey Cottage Hospital, £600, of which £500 to extension, in accordance with the scheme submitted to the Fund. Hospital for Consumption, Brompton (including Sanatorium at Frimley), £5,000. Hospital for Diseases of the Throat, £650, of which £500 towards loss incurred by postponement of building scheme through the war. Hospital for Epilepsy and Paralysis, £2,000, of which £500 to new out-patient department, in accordance with the scheme submitted to the Fund. Hospital for Sick Children, £2,650, of which £150 to fire staircase. Hospital for Women (Soho Square), £2,000, of which £5,000 to reduce debt. Hospital of St. John and St. Elizabeth, £500. Infants' Hospital, £800, of which £250 to reduce debt. Italian Hospital, £200. Kensington and Fulham General Hospital, £25. Kensington Dispensary and Children's Hospital, £25. King Edward Memorial Hospital (Ealing), £700, of which £5C to fire-escape staircase. King's College Hospital, £7,000, of which £2,000 to reduction of debt on removal to South London, making £79,100 in all contributed by the Fund to this purpose. London Fever Hospital, £250. London Homœopathic Hospital, £1,500. London Hospital, £13,000, of which £1,000 to deficit on new nurses' home, in accordance with the scheme submitted to the Fund. London Lock Hospital, £1,100, of which £1,000 to maintenance of Harrow Road Hospital and £100 to maintenance of Dean Street Hospital. London Temperance Hospital, £2,000, of which £1,000 to new nurses' home, in accordance with the scheme submitted to the Fund. Maternity Charity and District Nurses' Home, Plaistow, £1,300, of which £1,000 to rebuilding and extension, in accordance with the scheme submitted to the Fund. Memorial Hospital, Mildmay Park, £200. Metropolitan Hospital, £2,000. Middlesex Hospital, £5,000. Middlesex Hospital Cancer Charity, £500. Mildmay Mission Hospital, £550. Miller General Hospital for South-East London, £4,000, of which £1,500 to extension, in accordance with scheme to be submitted to the Fund. Mothers' Hospital of the Salvation Army, £850, of which £450 to new nurses' accommodation, in accordance with the scheme submitted to the Fund. National Hospital for Diseases of the Heart, £700, of which £200 to extension of out-patient department, in accordance with the scheme submitted to the Fund. National Hospital for the Paralysed and Epileptic, £3,000, of which £1,000 to reduce debt. Nelson Hospital (late South Wimbledon, etc.), £1,000, of which £500, to extension, in accordance with the scheme submitted to the Fund. Northcourt Hospital and Home for Sick Children, Hampstead, £50, in consideration of the fact that curable cases are admitted. Norwood Cottage Hospital, £450, of which £50 to $x$-ray and other improvements. Paddington Green Children's Hospital, £750, of which £250 to scheme. Passmore Edwards Hospital for Wood Green, etc., £75. Poplar Hospital for Accidents, £1,100, of which £1,000 to new casualty department, in accordance with the scheme submitted to the Fund. Prince of Wales's General Hospital, £5,000, of which £1,000 to reduce general fund debt and £2,000 to extension, in accordance with scheme to be submitted to the Fund. Queen Charlotte's Lying-in Hospital, £1,600, of which £850 to improved out-patient department, nurses' quarters, etc., in accordance with the scheme submitted to the Fund. Queen Mary's Hospital for the East End, incorporating West Ham and Eastern General Hospital, £6,000, of which £1,500 to new maternity block, in accordance with the scheme submitted to the Fund, and £1,000 to new nurses' home and operating theatre, in accordance with schemes to be submitted. Queen's Hospital for Children, £1,500. Royal Chest Hospital (City Road), £1,000. Royal Dental Hospital of London, £250. Royal Eye Hospital, £500. Royal Free Hospital, £4,500, of which £1,000 to new nurses' accommodation, in accordance with the scheme submitted to the Fund. Royal Hospital, Richmond, £400. Royal London Ophthalmic Hospital, £2,500, of which £500 to reduce debt. Royal National Orthopædic Hospital, £2,500, of which £1,000 to extension of out-patient department, in accordance with the scheme submitted to the Fund. Royal Waterloo Hospital for Children and Women, £3,000, of which £1,000 to new nurses' accommodation, isolation

**King Edward's Hospital Fund for London—(*continued*).**

wards, and extension of out-patient department, in accordance with the scheme submitted to the Fund. Royal Westminster Ophthalmic Hospital, £350, of which £50 to recent improvements in nurses' quarters. St. Andrew's Hospital (Dollis Hill), £150, of which £50 to improvements. St. Columba's Hospital, £250, of which £50 to provision of shelter to top balcony. St. George's Hospital, £7,000. St. John's Hospital, Lewisham, £750, of which £250 to new out-patient department, in accordance with the scheme submitted to the Fund. St. John's Hospital for Diseases of the Skin, £250, of which £50 to new nurses' accommodation, in accordance with the scheme submitted to the Fund. St. Luke's Hospital for Advanced Cases, £200. St. Mark's Hospital, £450, of which £50 to improvements in nursing accommodation. St. Mary's Hospital, £7,000. St. Mary's Hospital for Women and Children, Plaistow, £1,700, of which £500 to the rebuilding of the nurses' and servants' quarters and out-patient department, in accordance with the scheme submitted to the Fund. St. Monica's Home Hospital, £150. St. Peter's Hospital for Stone, £300. St. Saviour's Hospital for Ladies of Limited Means (Osnaburgh Street), £200. St. Thomas's Hospital, £11,250, of which £250 to lifts and £2,000 to enlargement of nurses' dining-room, in accordance with the scheme submitted to the Fund. Samaritan Free Hospital for Women, £1,300. Santa Claus Home, £50. South-Eastern Hospital for Children (Sydenham), £550, of which £250 to enlarged out-patient department, in accordance with the scheme submitted to the Fund. South London Hospital for Women, £1,000. Stoke Newington Home Hospital for Women, £75. University College Hospital, £6,000. Victoria Hospital for Children, £1,250. Walthamstow, Wanstead, and Leyton Children's and General Hospital, £600. West End Hospital for Nervous Diseases, £750. Western Ophthalmic Hospital, £400. West London Hospital, £2,000. Westminster Hospital, £2,800, of which £300 annual grant to acquisition of new site as agreed. Willesden Hospital, £1,850, of which £1,500 to extension as general hospital, in accordance with scheme to be submitted to the Fund. Wimbledon Hospital, £400, of which £250 to new nurses' home, in accordance with the scheme submitted to the Fund. Winifred House Invalid Children's Convalescent Hospital Home, £25. Woolwich and Plumstead Cottage Hospital, £25 (see special grant below). Total, £187,000.

Special Grant.

| | |
|---|---|
| Woolwich and District War Memorial Hospital Building Fund ... ... ... ... ... ... | £3,000 |

(to provision of general hospital, in accordance with scheme to be submitted to the King's Fund).

Summary.

| | |
|---|---|
| Grants ... ... ... ... ... ... ... ... ... | £187,000 |
| Special Grant ... ... ... ... ... ... ... | 3,000 |
| Total Grants to Hospitals at Ordinary Distribution, December 14, 1920 ... ... ... ... ... | 190,000 |
| Total Grants at Emergency Distribution, July 5, 1920 ... ... ... ... ... ... ... ... | 250,000 |
| Total Grants to Hospitals for the year 1920 ... | £440,000 |

(apart from a further £250,000 distributed on July 23, 1920, on behalf of the British Red Cross Society and the Order of St. John of Jerusalem, in aid of schemes of extension or improvement at hospitals able and willing to treat ex-Service men).

